# Successful Treatment of Secondary Hypersomnia Due to Complex Post-Traumatic Stress Disorder with Eye Movement Desensitization and Reprocessing: A Case Report

**DOI:** 10.3390/clockssleep7030043

**Published:** 2025-08-15

**Authors:** Vlad Bucurescu, Laure Peter-Derex, Maria Livia Fantini, Benjamin Putois

**Affiliations:** 1 Faculty of Psychology, UniDistance Suisse, 3900 Brig, Switzerland; vlad.bucurescu@etu.unidistance.ch; 2 Sleep Medicine and Respiratory Disease Center, Croix-Rousse Hospital, CHU of Lyon, 103 Gd Rue de la Croix-Rousse, 69000 Lyon, France; laure.peter-derex@chu-lyon.fr; 3 Lyon 1 University, 43 Bd du 11 Novembre 1918, 69000 Lyon, France; 4 Lyon Neuroscience Research Center, CNRS UMR 5292, INSERM U1028, Lyon 1 University, 69000 Lyon, France; 5 Institute Pascal, CNRS, Clermont Auvergne INP, Université Clermont Auvergne, 49 bd François Mitterrand, 63000 Clermont-Ferrand, France; maria_livia.fantini@uca.fr; 6 Neurophysiology Department, University Hospital, 58 rue Montalembert, 63000 Clermont-Ferrand, France

**Keywords:** hypersomnolence, hypersomnia, hypersomnia associated with psychiatric disorders, traumatic disorders, complex post-traumatic stress disorder, eye movement desensitization and reprocessing, Multiple Sleep Latency Test

## Abstract

Hypersomnia may be classified as primary or secondary, with secondary hypersomnia arising from a variety of underlying causes. Thus, according to ICSD3-TR classification, the diagnosis of idiopathic hypersomnia (IH) is established based on (1) excessive daytime sleepiness (EDS); (2) electrophysiological findings including either a mean sleep latency of less than 8 min on the Multiple Sleep Latency Test (MSLT) or increased total sleep (≥11 h) on 24 h polysomnography; and (3) systematic elimination of other potential etiologies, including sleep deprivation, substances, and medical, psychiatric (notably mood disorders), or sleep disorders. Nevertheless, the clinical heterogeneity observed in IH fuels an ongoing debate, reflecting the limited understanding of its underlying pathophysiological mechanisms. This report describes the case of a patient presenting with a clinical and polysomnographic phenotype of IH (MSLT < 8 min). A comprehensive psychopathological evaluation was performed to explore the possibility of secondary hypersomnia, which revealed features consistent with complex post-traumatic stress disorder (c-PTSD). Psychotherapy focused on c-PTSD was administered with positive and objective results in hypersomnolence/EDS. This clinical improvement suggests a potential relationship between psychological trauma and hypersomnia, a connection that is rarely described in the literature and warrants further investigation. This case highlights the need for a comprehensive assessment of secondary factors, particularly complex trauma, even in the presence of a clinical and polysomnographic phenotype consistent with IH.

## 1. Introduction

According to the ICD-11 (International Classification of Diseases, 11th Revision) [1], hypersomnia is classified under sleep disorders and is characterized by excessive daytime sleepiness (EDS) and repeated episodes of sleep during the day. Patients may exhibit prolonged nocturnal sleep or unintended daytime sleep episodes occurring at inappropriate times. The etiology of hypersomnia is heterogeneous [2]. The ICD-11 therefore distinguishes between primary hypersomnia (e.g., narcolepsy, Kleine–Levin syndrome [3]) and secondary hypersomnia. These secondary forms warrant in-depth investigations as they may be associated with medical conditions including neurological disorders, etc. (e.g., Parkinson’s disease [4]); genetic disorders (e.g., Prader–Willi syndrome [5]); tumoral disorders (e.g., craniopharyngioma); traumatic brain injury (hypersomnia occurring 6–18 months post-injury [6,7]); post-infectious syndrome (e.g., mononucleosis [8]); syndrome following H1N1 vaccination [9]; metabolic or endocrine diseases [2]; other sleep disorders (e.g., breathing-related sleep disorders [2], periodic limb movement disorder [10]); menstruation [11,12]; insufficient sleep syndrome [13]; substances (e.g., alcohol [14]); or psychiatric disorders [15]. This list is not exhaustive. The majority of clinical and scientific investigations into hypersomnia associated with psychiatric disorders have primarily focused on mood disorders [15,16]. To our knowledge, only one study has examined psychiatric conditions beyond mood disorders in the context of hypersomnia [17]: six patients with somatoform disorder, four with anxiety disorder, and one with personality disorder.

In the absence of these comorbidities or criteria suggestive of narcolepsy, patients exhibiting mean sleep latencies of less than 8 min (International Classification of Sleep Disorders-3 criteria [18]) on the Multiple Sleep Latency Test (MSLT) are diagnosed with idiopathic hypersomnia (IH). Although by definition, IH is a diagnosis of exclusion, the heterogeneity of this disorder currently presents a challenge, as it remains a condition with poorly understood underlying pathophysiology. Moreover, a meta-analysis demonstrated that one-quarter of patients with psychiatric hypersomnolence had MSL below 8 min on the MSLT [15]. In addition, long-term follow-up of patients with IH suggests that although clinical symptoms remain overall stable over time, the underlying diagnosis may change in almost half of patients [19]. This observation underscores the need for caution when diagnosing IH and highlights the importance of conducting comprehensive psychopathological investigations.

This article presents the case of a patient initially diagnosed with IH following an exhaustive differential diagnostic workup and appropriate sleep examination (polysomnography (PSG), MSLT with mean sleep latency < 8 min). Subsequently, the patient underwent a comprehensive psychological evaluation, which led to the diagnosis of complex post-traumatic stress disorder (c-PTSD). Complex trauma refers to a chronic or repeated exposure to multiple forms of trauma, most often occurring during childhood or adolescence, including at least one interpersonal trauma, defined as intentionally perpetrated by another person (e.g., physical or sexual abuse). According to the ICD-11 [1], c-PTSD is characterized by three core symptom domains. The first domain includes classic PTSD symptoms such as re-experiencing the traumatic event (s) in the present, avoidance of trauma-related stimuli, and a persistent sense of heightened threat manifesting as hypervigilance or an exaggerated startle response. The second domain, affective dysregulation, encompasses difficulties in emotional regulation, ranging from heightened emotional reactivity to emotional numbing or dissociation. The third domain, disturbances in self-organization, involves a negative self-concept marked by feelings of worthlessness or shame, significant difficulties in sustaining interpersonal relationships, and problems with social functioning and interpersonal boundaries. We therefore investigated the potential association between c-PTSD and hypersomnolence.

This patient received targeted psychotherapeutic interventions, including imagery rescripting therapy (IRT) and eye movement desensitization and reprocessing (EMDR). The treatment specifically focused on alleviating her nightmares and processing traumatic memories. Post-therapeutic assessment using PSG and MSLT revealed a marked reduction in hypersomnolence, accompanied by significant improvements across multiple objective sleep-wake parameters.

## 2. Detailed Case Description

This clinical report describes the case of a 33-year-old married female patient with two children, who primarily presented with complaints of recurrent nightmares and EDS. The patient reported multiple instances of unintended sleep episodes, including episodes occurring during driving, one of which nearly resulted in a road accident.

### 2.1. Anamnesis

The patient described a developmental environment marked by severe emotional neglect. Her childhood was characterized by prolonged periods without adult supervision and an absence of household structure. Her brother, who exhibited chronic behavioral disturbances (including aggression, substance misuse, and criminal activity), was also the perpetrator of repeated intrafamilial sexual abuse. From the ages of 8 to 14, she was subjected to non-consensual sexual intercourse, which she never disclosed to her parents. This concealment was driven by fear of punishment, intense guilt, and dissociative defenses.

Her psychological response to this chronic trauma was notable for dissociative symptoms and pervasive internalized shame. The patient reported feeling “contaminated” and responsible for the abuse due to her silence, reflecting a core feature of c-PTSD: persistent negative self-concept. She retrospectively hypothesized that her hypersomnolence may have served as a functional dissociative strategy during adolescence—allowing her to avoid proximity to the perpetrator and regulate overwhelming affective states. Daytime sleep became a means of psychological withdrawal in the context of an environment from which no physical escape was possible.

Parental relationships were marked by emotional unavailability. The mother was described as cold, emotionally disengaged, and frequently demeaning toward the father, who, in turn, was passive and affectively distant. The patient recalled never having felt able to confide in either parent and reported no protective adult figures present during her development.

At age 25, the patient began to experience a reactivation of post-traumatic symptoms, including nightmares and EDS, following exposure to occupational stress in a male-dominated profession (the police force) and during her first marital relationship. Although the nightmare content varied, the underlying affective tone was consistently marked by insecurity and threat. She also experienced postpartum depression following the birth of each child, which responded favorably to antidepressant treatment and subsequently remitted.

Additionally, the patient had presented with iron deficiency (ferritin <30 ng/mL) secondary to heavy menstrual bleeding, which led to intermittent symptoms of restless legs syndrome. These symptoms were appropriately treated and did not account for the severity of her hypersomnolence. An otolaryngological examination noted pathological nocturnal jaw clenching.

### 2.2. Evaluation and Diagnostic

The patient reported frequent nightmares—ranging from one to three episodes per night—characterized by recurring themes of insecurity and natural disasters (e.g., tornadoes, fires, tsunamis). She also reported regularly taking dreamless daytime naps, which she subjectively experienced as more restorative than nocturnal sleep. Despite an estimated total nocturnal sleep duration of approximately 8.5 h, she complained of persistent EDS, scoring 19 out of 24 on the Epworth Sleepiness Scale.

The patient presented with pervasive feelings of guilt and inner contamination, accompanied by significant autonomic arousal, including sustained muscle tension, generalized anxiety, and chronic fatigue. She described persistent mental rumination, with recurring intrusive cognitions such as: “I am overwhelmed,” “I have no way out,” “I am unsafe,” and “I am stuck.” She reported sexual dysfunction marked by dissociative symptoms during intercourse (including derealization and detachment from bodily sensations), anorgasmia, and guilt associated with the prospect of refusing her husband’s sexual advances. She also exhibited hypersensitivity to human-generated sounds and a pervasive sense of physical and psychological insecurity. Under stress, she reported frequent irritability and disproportionate emotional reactivity to minor stimuli.

Anamnestic screening for narcolepsy was negative: the patient denied experiencing cataplexy, hypnagogic or hypnopompic hallucinations, and sleep paralysis. Neurological examination revealed no abnormalities. The patient completed a sleep diary (recorded over a 14-day period) to rule out chronic sleep deprivation or circadian misalignment before PSG. The total sleep time was 8 h and 17 min, with an average bedtime of 10:42 pm and wake time of 7:07 am. Sleep efficiency was 95.6%. The patient took a non-refreshing nap of approximately 35 min daily and reported an average of four episodes of EDS per day. Overnight PSG demonstrated normal sleep architecture and the absence of respiratory disturbances (see Table 1). However, the MSLT revealed a pathological mean sleep latency of 3 min, without sleep-onset REM periods (SOREMPs). Based on these findings, a provisional diagnosis of IH was established.

In parallel, the psychological evaluation identified a severe form of c-PTSD, with marked dissociative symptoms.

Despite her psychopathological history, the patient described herself positively, endorsing traits such as reliability, attentiveness, helpfulness, and humor. She identified her core values as honesty, transparency, mutual support, and kindness.

### 2.3. Treatments

The patient was subsequently referred to a psychotherapist (last author) for a non-pharmacological intervention. Psychotherapy was conducted in two phases. The initial phase consisted of six sessions of IRT [24], a modality recommended for the treatment of trauma-related nightmares. This intervention yielded clinically significant improvements, including a marked reduction in nightmare frequency and enhanced subjective sleep quality.

Despite these gains, residual symptoms persisted, notably EDS, sexual dysfunction, unresolved shame, and unexpressed anger. The positive outcomes achieved in the first phase contributed to the reinforcement of the patient’s sense of self-efficacy and consolidated the therapeutic alliance, thereby motivating her to engage in deeper trauma-focused work.

The second phase of treatment comprised twelve sessions of EMDR [25]. During these sessions, the patient processed core traumatic memories from childhood, expressed repressed anger and indignation, and was able to reframe her guilt with greater psychological distance. She demonstrated a high level of compliance and engagement throughout both phases of therapy. Importantly, no specific instructions or interventions targeting sleep behavior were provided at any point during the psychotherapeutic treatment.

## 3. Results

Following the two-phase trauma-focused psychotherapy, both chronic nightmares and symptoms of c-PTSD were in full remission (see Table 1). A follow-up overnight PSG and MSLT were conducted 19 months after the initial assessment. Notably, although no direct intervention targeting hypersomnolence was implemented, the psychotherapeutic intervention led to a marked reduction in both subjective daytime sleepiness, as measured by the Epworth Sleepiness Scale, and objective hypersomnolence, as evidenced by improved MSLT findings. The nocturnal polysomnographic results were approximately equivalent between the pre-treatment and post-treatment assessments; however, mean sleep latency on the MSLT increased from 3 min to 16.6 min. Comparison of sleep diary data revealed stable nocturnal sleep parameters, along with a marked reduction in both EDS and nap duration. Post-treatment sleep diary data indicated a total sleep time of 8 h and 12 min, with a mean bedtime of 10:52 pm, wake time of 7:40 am, and a sleep efficiency of 96.4%. The patient reported a single nap per day lasting approximately 13 min and experienced one daily episode of EDS, typically occurring around 1:00 pm. Additionally, marital distress, guilt, and irritability had significantly decreased. It is also noteworthy that the patient no longer experienced nocturnal awakenings or nightmares, reflecting a substantial improvement in insomnia-related symptoms.

## 4. Discussion

The main observation of this case is the marked reduction in EDS following remission of psychological trauma, in line with the hypothesis that c-PTSD was responsible for the symptoms. This suggests that traumatic etiological factors may underlie some cases of hypersomnia presenting with a polysomnographic phenotype consistent with IH.

To our knowledge, the relationship between hypersomnia and traumatic disorders remains unexplored in the current literature. While numerous studies within sleep medicine have established links between sleep disturbances and psychological trauma, none have specifically examined EDS or hypersomnia [26,27,28]. Likewise, research addressing the phenomenology of PTSD [29] or adverse childhood experiences (defined as traumatic events occurring before 18 years of age) [30] has documented associations with sleep disturbances, but has not focused on hypersomnolence.

### 4.1. Limitations

This single case study has inherent limitations. It would have been valuable to perform PSG and MSLT between the two phases of psychotherapy to better characterize changes over time. However, it should be noted that IRT primarily reduced nightmares and nocturnal awakenings but had a limited impact on EDS; moreover, IRT is not considered a first-line treatment for c-PTSD. Unfortunately, the diagnostic evaluation of this case did not include actigraphy or circadian assessment. It is possible that the patient was sleep-deprived or had sleep–wake patterns misaligned with her circadian rhythm. However, it should be noted that both the duration and efficiency of her sleep remained stable between the pre- and post-treatment periods, while her EDS markedly decreased. Objective measurements of these rhythms prior to the PSG would have strengthened the observations derived from the sleep diary. Assessments of dissociative symptoms and attachment disorders would also have been relevant, but were not routinely conducted in our clinic. Given the single-subject design, caution is required in generalizing these findings. Larger-scale studies are necessary to substantiate and expand upon these preliminary observations. Nonetheless, this case highlights a novel potential link between c-PTSD and hypersomnia, opening new avenues for future research.

### 4.2. Clinical Implications

From a clinical perspective, this report highlights the critical importance of conducting a thorough psychological evaluation in patients presenting with EDS or suspected IH. Even among individuals who fulfil the ICSD-3 diagnostic criteria for IH, comorbid or underlying psychiatric disorders can substantially influence the clinical presentation or mimic primary central hypersomnolence disorders. This case advocates for a multidisciplinary approach, emphasizing the necessity of integrating comprehensive psychological assessment and tailored therapeutic interventions into the standard management of hypersomnolence. In the clinical evaluation of hypersomnia, c-PTSD should be systematically assessed through a comprehensive psychological evaluation, and appropriate targeted treatment should be offered. However, identifying c-PTSD may be challenging for clinicians who lack specialized training in psychotraumatology, as patients often present with dissociative symptoms, avoidance behaviors, or denial. To address these challenges within sleep medicine, it is essential to obtain a thorough medical and psychosocial history when assessing hypersomnia. Several validated instruments can facilitate this evaluation, including the Trauma History Questionnaire (THQ) [31], which comprises 24 items assessing exposure to a broad range of traumatic events; the Childhood Trauma Questionnaire (CTQ) [32], a 28-item inventory evaluating childhood abuse and neglect; the International Trauma Questionnaire (ITQ) [33], an 18-item measure that assesses diagnostic criteria for PTSD and c-PTSD; the Dissociative Experiences Scale (DES) [34], a 28-item scale quantifying dissociative symptoms and their daily frequency; and the Somatoform Dissociation Questionnaire (SDQ-20) [35], a 20-item instrument measuring the severity of somatoform dissociative manifestations. Incorporating these tools into the diagnostic process may improve detection of c-PTSD and facilitate more personalized management strategies in patients with a polysomnographic phenotype consistent with IH.

## 5. Conclusions

In conclusion, this clinical case reinforces the potential association between hypersomnia and traumatic disorders. We hypothesize that hypersomnolence may represent a dissociative response to inescapable, repeated trauma, particularly in cases involving complex etiologies such as childhood abuse, where avoidance or confrontation is not possible. Should these findings be substantiated by further empirical research, they could pave the way for novel therapeutic approaches in certain cases of hypersomnia, including trauma-focused interventions such as Cognitive Behavioral Therapy with prolonged exposure or EMDR, which are currently recommended treatments for complex PTSD [21].

## Figures and Tables

**Table 1:** Evolution of symptoms between pre- and post-treatment recorded with polysomnography (PSG) and questionnaires.

Variables and Measures	Pre-Treatment	Post-Treatment
14-day Sleep diary—Total Sleep Time	8 h17	8 h12
14-day Sleep diary—Sleep Efficiency	95.6%	96.4%
14-day Sleep diary—Nap Duration	35 min	13 min
**MLST—Multiple Sleep Latency Test mean latency**	**3 min**	**16.6 min**
PSG—Total Sleep Duration	450 min	399.5 min
PSG—Sleep Onset Latency	2.5 min	9.5 min
PSG—Sleep Efficiency	94.9%	82.9%
PSG—Awakenings	21	23
PSG—% N1	9.5%	8.%
PSG—% N2	48.4%	45.3%
PSG—% N3	16%	12.2%
PSG—% REM	21.5%	18.6%
PSG—% wake	4.6%	15.5%
PSG—N1 Latency	2.5 min	9.5 min
PSG—N2 Latency	4.0 min	12.5 min
PSG—N3 Latency	14.5 min	20.0 min
PSG—Latency to stage R from sleep onset	107.5 min	115 min
PSG—arousals	numbers: 86/index: 11.5	Numbers: 71/index: 10.7
**EPWORTH—Severity of Somnolence (Epworth** [20]**)**	**19**	**5**
ISI—Severity of Insomnia (Insomnia Severity Index [21])	11	11
BIS—Frequency of Insomnia (Bergen Insomnia Scale [22])	21	11
**Average number of nightmares per week (Sleep Diary)**	**13**	**0.5**
**Severity of Post-Traumatic Stress Disorder (PCL-S 5** [23]**)**	**48**	**7**

## Abbreviations

The following abbreviations are used in this manuscript:

c-PTSD	complex post-traumatic stress disorder
EDS	excessive daytime sleepiness
EMDR	eye movement desensitization and reprocessing
ENT	otorhinolaryngologist
IRT	imagery rescripting therapy
MLST	Multiple Sleep Latency Test
PSG	polysomnography
REM	rapid eye movement
SOREMP	sleep onset rapid eye movement periods

## References

[1] World Health Organization (2018). International Classification of Diseases 11th Revision.

[2] Dauvilliers Y., Buguet A. (2005). Hypersomnia. Dialogues Clin. Neurosci..

[3] Miglis M.G., Guilleminault C. (2014). Kleine-Levin Syndrome: A Review. Nat. Sci. Sleep.

[4] Arnulf I. (2005). Excessive Daytime Sleepiness in Parkinsonism. Sleep Med. Rev..

[5] Ghergan A., Coupaye M., Leu-Semenescu S., Attali V., Oppert J.-M., Arnulf I., Poitou C., Redolfi S. (2017). Prevalence and Phenotype of Sleep Disorders in 60 Adults With Prader-Willi Syndrome. Sleep.

[6] Billiard M., Podesta C. (2013). Recurrent Hypersomnia Following Traumatic Brain Injury. Sleep Med..

[7] Imbach L.L., Valko P.O., Li T., Maric A., Symeonidou E.-R., Stover J.F., Bassetti C.L., Mica L., Werth E., Baumann C.R. (2015). Increased Sleep Need and Daytime Sleepiness 6 Months after Traumatic Brain Injury: A Prospective Controlled Clinical Trial. Brain J. Neurol..

[8] Sforza E., Hupin D., Roche F. (2018). Mononucleosis: A Possible Cause of Idiopathic Hypersomnia. Front. Neurol..

[9] Berntsson S.-G., Sarkanen T., Saric A., Alakuijala A., Partinen M., Landtblom A.-M. (2025). Post-Vaccination Hypersomnia—Occurrence of Idiopathic Hypersomnia and Narcolepsy Type 2 after Pandemrix Vaccination in a Case- Series from Sweden and Finland. J. Neurol. Sci..

[10] Nicolas A., Lespérance P., Montplaisir J. (1998). Is Excessive Daytime Sleepiness with Periodic Leg Movements during Sleep a Specific Diagnostic Category?. Eur. Neurol..

[11] Suau G.M., Cabrera V., Romaguera J. (2016). Menstruation-Related Hypersomnia Treated with Hormonal Contraception: Case Report and Review of Literature. Puerto Rico Health Sci. J..

[12] Sachs C., Persson H.E., Hagenfeldt K. (1982). Menstruation-Related Periodic Hypersomnia: A Case Study with Successful Treatment. Neurology.

[13] Ohayon M.M. (2008). From wakefulness to excessive sleepiness: What we know and still need to know. Sleep Med Rev..

[14] Papineau K.L., Roehrs T.A., Petrucelli N., Rosenthal L.D., Roth T. (1998). Electrophysiological Assessment (The Multiple Sleep Latency Test) of the Biphasic Effects of Ethanol in Humans. Alcohol. Clin. Exp. Res..

[15] Plante D.T. (2017). Sleep Propensity in Psychiatric Hypersomnolence: A Systematic Review and Meta-Analysis of Multiple Sleep Latency Test Findings. Sleep Med. Rev..

[16] Billiard M., Dolenc L., Aldaz C., Ondze B., Besset A. (1994). Hypersomnia Associated with Mood Disorders: A New Perspective. J. Psychosom. Res..

[17] Vgontzas A.N., Bixler E.O., Kales A., Criley C., Vela-Bueno A. (2000). Differences in Nocturnal and Daytime Sleep between Primary and Psychiatric Hypersomnia: Diagnostic and Treatment Implications. Psychosom. Med..

[18] American Academy of Sleep Medicine (2014). International Classification of Sleep Disorders.

[19] Šonka K., Feketeová E., Nevšímalová S., Horvat E.M., Příhodová I., Dostálová S., Galušková K., Milata M., Bušková J., Susta M. (2024). Idiopathic Hypersomnia Years after the Diagnosis. J. Sleep Res..

[20] Johns M.W. (1991). A New Method for Measuring Daytime Sleepiness: The Epworth Sleepiness Scale. Sleep.

[21] Morin C.M., Belleville G., Bélanger L., Ivers H. (2011). The Insomnia Severity Index: Psychometric Indicators to Detect Insomnia Cases and Evaluate Treatment Response. Sleep.

[22] Pallesen S., Bjorvatn B., Nordhus I.H., Sivertsen B., Hjørnevik M., Morin C.M. (2008). A New Scale for Measuring Insomnia: The Bergen Insomnia Scale. Percept. Mot. Skills.

[23] Blanchard E.B., Jones-Alexander J., Buckley T.C., Forneris C.A. (1996). Psychometric Properties of the PTSD Checklist (PCL). Behav. Res. Ther..

[24] Aurora R.N., Zak R.S., Auerbach S.H., Casey K.R., Chowdhuri S., Karippot A., Maganti R.K., Ramar K., Kristo D.A., Standards of Practice Committee (2010). Best Practice Guide for the Treatment of Nightmare Disorder in Adults. J. Clin. Sleep Med..

[25] (2019). Guideline Development Panel for the Treatment of PTSD in Adults, Summary of the Clinical Practice Guideline for the Treatment of Posttraumatic Stress Disorder (PTSD) in Adults. Am. Psychol..

[26] Spoormaker V.I., Montgomery P. (2008). Disturbed Sleep in Post-Traumatic Stress Disorder: Secondary Symptom or Core Feature?. Sleep Med. Rev..

[27] Ohayon M.M., Shapiro C.M. (2000). Sleep Disturbances and Psychiatric Disorders Associated with Posttraumatic Stress Disorder in the General Population. Compr. Psychiatry.

[28] Kobayashi I., Boarts J.M., Delahanty D.L. (2007). Polysomnographically Measured Sleep Abnormalities in PTSD: A Meta-Analytic Review. Psychophysiology.

[29] van der Kolk B.A., Roth S., Pelcovitz D., Sunday S., Spinazzola J. (2005). Disorders of Extreme Stress: The Empirical Foundation of a Complex Adaptation to Trauma. J. Trauma. Stress.

[30] Kajeepeta S., Gelaye B., Jackson C.L., Williams M.A. (2015). Adverse Childhood Experiences Are Associated with Adult Sleep Disorders: A Systematic Review. Sleep Med..

[31] Hooper L.M., Stockton P., Krupnick J.L., Green B.L. (2011). Development, Use, and Psychometric Properties of the Trauma History Questionnaire. J. Loss Trauma.

[32] Bernstein D.P., Fink L., Handelsman L., Foote J., Lovejoy M., Wenzel K., Sapareto E., Ruggiero J. (1994). Initial Reliability and Validity of a New Retrospective Measure of Child Abuse and Neglect. Am. J. Psychiatry.

[33] Cloitre M., Hyland P., Prins A., Shevlin M. (2021). The International Trauma Questionnaire (ITQ) Measures Reliable and Clinically Significant Treatment-Related Change in PTSD and Complex PTSD. Eur. J. Psychotraumatology.

[34] Bernstein E.M., Putnam F.W. (1986). Development, Reliability, and Validity of a Dissociation Scale. J. Nerv. Ment. Dis..

[35] Nijenhuis E.R., Spinhoven P., Van Dyck R., Van der Hart O., Vanderlinden J. (1996). The Development and Psychometric Characteristics of the Somatoform Dissociation Questionnaire (SDQ-20). J. Nerv. Ment. Dis..

